# Ovarian Follicular Response Is Altered by Salpingectomy in Assisted Reproductive Technology: A Pre- and Postoperative Case–Control Study

**DOI:** 10.3390/jcm12154942

**Published:** 2023-07-27

**Authors:** Laurianne Reitz, Vincent Balaya, Basile Pache, Anis Feki, Grégoire Le Conte, Achraf Benammar, Jean-Marc Ayoubi

**Affiliations:** 1 Obstetrics and Gynecology Department, Foch Hospital, 92150 Suresnes, France; g.le-conte@hopital-foch.com (G.L.C.); a.benammar@hopital-foch.com (A.B.); jm.ayoubi@hopital-foch.com (J.-M.A.); 2 Gynecology and Obstetrics Unit, University Hospital of Reunion Island, BP 350, 97448 Saint Pierre Cedex, France; 3 Department of Obstetrics and Gynecology, Félix Guyon Hospital, CHU Nord of La Réunion, 97400 Saint-Denis, France; vincent.balaya@chu-reunion.fr; 4 Obstetrics and Gynecology Department, Fribourg Cantonal Hospital, 1708 Fribourg, Switzerland; basile.pache@h-fr.ch (B.P.); anis.feki@h-fr.ch (A.F.); 5 Faculty of Medicine, University of Fribourg, 1700 Fribourg, Switzerland; 6 Medical School, University of Versailles Saint-Quentin, 78000 Versailles, France

**Keywords:** assisted reproductive technology, in vitro fertilization, ovarian stimulation, follicles count, salpingectomy

## Abstract

***Objectives:*** The goal of this study was to assess the effect of unilateral salpingectomy on the number of mature follicles in the ipsilateral ovary during an assisted reproductive technology (ART) stimulation cycle, as compared to the contralateral ovary. ***Methods:*** This was a retrospective, single-center, case–control cohort study conducted from 2017 to 2022. Patients from 18 to 43 years old who underwent at least one ART cycle before and after a unilateral salpingectomy were included. The number of recruited follicles, including mature (≥16 mm) and intermediate follicles (13–15.5 mm), on the salpingectomy side (case) were compared to those present on the contralateral ovary (control) during an ART attempt. To take into account the inter-ovarian variability, the comparison was performed on two ART cycles, performed before then after the salpingectomy. ***Results:*** Overall, 24 patients were included in our study. While the number of mature follicles was similar in both ovaries before surgery, the mean number of mature follicles was significantly reduced after salpingectomy in the operated side, as compared to the control side, being, respectively 3.00 vs. 5.08 (*p* = 0.048). There was no significant difference between the intermediate and total recruited follicles. ***Conclusions:*** Our study suggests that salpingectomy may impact the follicle recruitment on the ipsilateral side by altering the vascularization during mesosalpinx coagulation. Gynecologists should be mindful of this concept and accurately set surgical indications. Beyond the indication, this emphasizes the critical role of having infertility surgeons sensitive to fertility preservation for optimal management of ART patients. Further studies with larger patient populations are required to confirm these results.

## 1. Introduction

Unilateral salpingectomy, which consists of the removal of all the fallopian tubes from the uterine horn to the pavilion, is a common surgical procedure in gynecology. Its indications include ectopic pregnancies with a damaged tube [1], permanent contraception by bilateral salpingectomy [2], or, more recently, prophylactic measure to reduce the risk of epithelial ovarian cancer [3]. In assisted reproductive technology (ART), it is also indicated in cases of hydrosalpinx. Indeed, hydrosalpinx consists of a collection of serous or clear fluid blocked in the fallopian tubes due to distal tubal occlusion, primarily post-infectious. This condition, which can reach a frequency of 30% in women with tubal infertility [4], results in a significant reduction in implantation and pregnancy rates, as well as an increased miscarriage rate in patients undergoing embryo transfer as compared to those without hydrosalpinx [5,6,7,8,9]. Therefore, its removal is recommended before embryo transfer [10,11]. Considering that 30% of female infertility is linked to tubal factors [12,13], this surgical procedure is commonly performed before or during ART management.

However, due to the anatomical links between the fallopian tubes and the ovary, the surgical procedure of salpingectomy may cause some side effects on ovarian function. In fact, these two organs share a common vascularization, with a communicating arcade between the uterine and ovarian arteries through the mesosalpinx [14]. Coagulation and hemostasis during surgery may potentially alter the blood flow to the ipsilateral ovary. This raises concerns about salpingectomy on ovarian response in subsequent ART cycles.

Over the past thirty years, the impact of this surgery has been assessed by several parameters, including antral follicle count (AFC), hormone levels (follicle-stimulating hormone (FSH) and anti-mullerian hormone (AMH)), or gonadotropins administration dose, stimulation cycle length, oocyte retrieval, and pregnancy rates, as highlighted by these eight systematic reviews and meta-analyses [15,16,17,18,19,20,21,22]. Although its impact has been demonstrated in animal studies [23,24], results in human remain controversial due to several biases, as inter-individual and intra-individual—inter-cycle—variabilities in ovarian responses [25]. Previous studies have compared different ART cycles without taking to consideration the age variable, therefore potentially establishing bias in terms of ovarian response. Also, we believed that those studies who compared their cohort with control group that had no history of salpingectomy were far from ideal in assessing the impact of salpingectomy.

Prompted by the limitations that hampered previous research, we hypothesized that comparing the ovary on the operated side (case) to the non-operated side from the same patient (control), before and after surgery, would limit the impact of confounding factors.

The aim of this study was to assess the impact of unilateral salpingectomy on the ovarian response to hormonal hyperstimulation, as reflected by the number of mature follicles at induction (diameter ≥ 16 mm, according to the ESHRE guidelines [26]) in an ART cycle, before and after unilateral salpingectomy, compared to the contralateral ovary in the same patient.

## 2. Material and Methods

### 2.1. Patient Selection

A retrospective, observational, monocentric, case–control pre- and postoperative study was conducted based on the database of the ART department of the University Hospital of Foch (Suresnes, France), from 1 January 2017 to 30 November 2022. Data were collected from the hospital software database (Medifirst^®^).

Inclusion criteria were patients aged 18 to 43 years who underwent at least one in vitro fertilization (IVF) cycle with or without intra-cytoplasmic sperm injection (ICSI) before and after a unilateral salpingectomy. Exclusion criteria were bilateral salpingectomy and a history of previous tubal or ovarian surgeries (fimbrioplasty, ovariectomy, cystectomy, or surgical treatment of endometrioma), which have been shown to alter ovarian function.

Approval to conduct this study was obtained from the local ethical board (IRB000012437).

### 2.2. Protocol Characteristics

ART treatment followed our routine protocols. Ovarian hyperstimulation was achieved using highly purified gonadotropins: recombinant FSH, urinary FSH, and/or menotropins. Individually set doses of hormones were used, ranging from 150 to 600 IU of FSH per day in an antagonist protocol or a long agonist protocol. Development of ovarian follicles was monitored by manually calculating the average of the two largest diameters using a 5.0–9.0-MHz multifrequency transvaginal ultrasonography probe (Voluson™ S8 system, GE Healthcare, Chicago, IL, USA) and hormonal examinations starting on the 6th day of ovarian stimulation. If necessary, hormonal doses were adjusted to generate an optimal response. In case of antagonist protocol, daily antagonist was systematically introduced from the 6th day of hormonal stimulation. Final oocyte maturation was typically induced when ≥3 mature follicles (16–22 mm in diameter) were observed and estradiol levels per pre-ovulatory follicle were >200 pg/mL.

Endpoints measurements included the number of follicles on each ovary visualized by sonography on the day of triggering for each IVF attempt (both before and after the unilateral salpingectomy). The primary endpoint was the number of mature follicles ≥16 mm on the day of ovarian triggering, according to the 2019 ESHRE guidelines [26]. The secondary endpoints were the number of intermediate follicles (13 to 15.5 mm in diameter) and the total of recruited follicles (intermediate and mature). Subgroups analysis were conducted according to the indication of salpingectomy, specifically hydrosalpinx or ectopic pregnancy.

### 2.3. Data Analysis

Age at both IVF attempts, body mass index (BMI), antral follicles count (AFC), primary or secondary infertility and etiology, side of the salpingectomy, delay between surgery and postoperative IVF, protocol and dose of gonadotropins used, stimulation length, and hormone levels (FSH and AMH) were extracted. Poor ovarian reserve was defined by an AMH level < 1.00 ng/mL, FSH level > 10 UI/L, or an AFC < 4 follicles in both ovaries. Ovulatory dysfunction was characterized by irregular or absent menstrual cycles. The study population was divided into two subgroups based on the indication for salpingectomy, hydrosalpinx, or ectopic pregnancy.

### 2.4. Statistical Analysis

Qualitative variables were expressed as n (%) and were compared by applying Chi-square test. Quantitative variables were expressed as mean (range) or means ± SDs and were compared by applying T-Student test or by the Wilcoxon Mann–Whitney test in case of non-parametric distribution. Parametric distribution was tested using the Shapiro–Wilk test. All statistical tests were two-sided, and *p*-values lower than 0.05 were retained as a significance set. All statistical tests were carried out using R Studio (Version 1.2.5042, The R Foundation for Statistical Computing, Vienna, Austria).

## 3. Results

### 3.1. Population Characteristics

During the study period, 48 patients were recruited overall the 4945 women treated in our ART unit. We excluded 15 patients since they had a bilateral salpingectomy and 9 more that had another tubal or ovarian surgery. Finally, 24 patients were included. Salpingectomy was performed in 15 cases (62.5%) due to ectopic pregnancy, and in 9 cases (37.5%) due to hydrosalpinx. The mean BMI was 26.1 ± 5.6 kg/m^2^. Among etiologies of infertility, 45.8% of patients had tubal infertility and 41.7% had endometriosis. Poor ovarian reserve was detected in 29.2% of patients. Ovulatory dysfunction, most represented by the polycystic ovary syndrome (PCOS), was observed in 16.7% of patients. A male factor was found in 29.2% of cases. All salpingectomies were performed by minimally invasive surgery: 41.7% on the right side and 58.3% on the left side (Table 1A). Among the 24 salpingectomies, 18 were performed using bipolar energy, whereas six were not specified.

Clinical characteristics of both IVF cycles before and after salpingectomy are shown in Table 1B. No significant difference was observed between the two IVFs cycle except for the total dose of gonadotropins used during stimulation. The mean age of the patients was 34.9 ± 4.0 years during their first IVF and 36.8 ± 4.0 years during their second IVF attempt. Salpingectomy was performed in the interval between the first and the second ART cycle.

### 3.2. Comparison Control Side to Salpingectomy Side

There was no difference in ovarian response after stimulation for IVF between the two ovaries prior to salpingectomy (IVF number 1), with 4.00 and 3.79 mature follicles, respectively (*p* = 0.76). However, after salpingectomy (IVF number 2), the number of mature follicles on the day of the trigger was significantly lower in the ipsilateral ovary compared to the contralateral ovary, at 3.00 and 5.08 follicles, respectively (*p* = 0.048). No significant difference was found when analyzing the intermediate follicles during the first IVF (1.83 versus 1.65, *p* = 0.98) or during the second IVF (1.38 versus 1.21, *p* = 0.90). The total number of recruited follicles was not significantly different on both IVFs (5.74 versus 5.36, *p* = 0.69 for the first IVF and 4.38 versus 6.23, *p* = 0.13 for the second IVF). Results are shown in Table 2.

### 3.3. Subgroup Analysis

In the subgroup who underwent salpingectomy for ectopic pregnancy, no significant difference was observed before surgery with 4.67 versus 4.40 mature follicles on the salpingectomy and control sides on the day of hCG triggering (*p* = 0.85) and also after surgery with 3.90 versus 5.53 mature follicles (*p* = 0.22). The average time between surgery and the post-operative stimulation cycle was 11.8 months. Detailed results can be found in Table 3A.

In the subgroup who underwent salpingectomy for hydrosalpinx, no significant difference was observed in the ovarian response prior to surgery, with 2.89 follicles on the hydrosalpinx side compared to 2.78 follicles on the control side (*p* = 0.85) and after surgery with 1.67 mature follicles recruited on the salpingectomy side versus 4.33 mature follicles in the healthy side (*p* = 0.19). The average time between surgery and the post-operative stimulation cycle was 13.4 months. Detailed results are presented in Table 3B.

## 4. Discussion

Our results revealed that salpingectomy could impact ovarian response to ovarian stimulation in ART cycles, as reflected by the reduced number of mature follicles on the ipsilateral ovary at the time of hCG administration following surgery. Indeed, the ovarian response on the salpingectomy side was significantly reduced compared to the contralateral one, whereas both ovaries had a similar response before surgery. Furthermore, no difference was observed between the two IVFs cycle characteristics. To our knowledge, this was the first study combining the two designs of comparing the ovary “before-after surgery” and “operated adnexa versus contralateral side” in the same patient. Twenty-four patients were included, making a total of 48 case–control ovaries. The apparent smallness of this number (48 patients out of the 16,200 in the center), even if it was equivalent to that of other studies conducted on the subject [27,28,29,30,31,32,33], was mainly explained by the rare events of the IVF–salpingectomy–IVF sequence. This extremely selective design was chosen in order to limit bias, even though we knew it would lower the number of eligible patients. Patients in the second IVF cycle were older with a slightly lower mean AFC, but these differences were not statistically significant. This did not constitute a bias because we never compared the two IVF cycles between them but each ovary to the other within the same cycle. The patient served as their own control, meaning both ovaries were subjected to the same confounding factors. Our results were consistent with other studies, such as Gelbaya et al.’s [34], who found a lower number of follicles in case of salpingectomy as compared with proximal tubal division or control group, or Lass et al.’s [30], who found fewer follicles developed from the ovary of the operated side compared to contralateral one in 29 patients. Finally, the present results were in agreement with those of Orvieto et al. [27], whose pre-post design did not allow the exclusion of a time-related effect.

In the ectopic pregnancy subgroup, there was a trend toward a decreased number of postoperative mature follicles, but it was not statistically significant (*p* = 0.22). In the hydrosalpinx subgroup, the same trend was observed without reaching a statistically significant set with only 1.67 follicles versus 4.33 on the healthy side. In these cases, the harmful effects of a blowout mesosalpinx, major adhesions, and a less well-defined cleavage plane might play a role. This lack of significance was likely due to the small sample size, which could imply a loss of statistical power. This trend remains intriguing since the aim of salpingectomy is to improve fertility rates in ART, and, surprisingly, it appears to reduce follicular response compared to the preoperative IVF, which did not show any difference in response between the two ovaries when the hydrosalpinx was present.

Anatomically, assessing follicles numbers and size during ovarian stimulation provides interesting information, as it likely reflects respective ovarian perfusion on the operated and contralateral ovary. The salpingectomy procedure involves coagulation and section of the mesosalpinx from the tubo-ovarian ligament to the uterine horn. This mesosalpinx houses vascular anastomoses between the uterine and ovarian arteries, which provide blood supply to the ovary. Care is needed during tubal resection to stay close to the tube to preserve as much mesosalpinx as reasonable. Preserving the mesosalpinx theoretically decreases the compromise of the mesosalpingeal blood supply to the ovary. Following salpingectomy, blood flow to the ipsilateral ovary might be altered either directly or through thermal injuries and diffusion, leading to a decrease in ovarian follicular mass or perfusion of gonadotropins [35,36]. The final maturation of follicles, beyond the antral stage, is dependent on FSH. When the follicle reaches a diameter of 10 mm, it becomes sensitive to FSH, and, under the action of FSH, the dominant follicle whose diameter is greater than 14 mm expresses LH receptors in the granulosa cells. If the ovary receives a reduced amount of FSH due to disrupted blood flow, the growth of follicles may be hindered. By increasing the doses of gonadotropins [18,20,37,38] or the duration of stimulation [38], the damage in blood supply may be, in part, compensated, explaining the absence of significant results in other studies in terms of total number of retrieved oocytes or pregnancy rates [15,16,17,18,22,28,29,30,31,32,33,37,38,39]. Ultimately, boosting the total dose (through daily dosage or duration) either allows for the recruitment of follicles from the operated side or enables the healthy ovary to recruit more mature follicles. This may account for the absence of a significant decrease in the intermediate follicles and the total number of recruited follicles observed in our study. Beyond an increase in duration or dosage of gonadotropins, some authors believe that the lack of difference implies the existence of compensatory mechanisms in the unaffected ovary [37].

Furthermore, it is possible that salpingectomy does not alter ovarian reserve, but only the ovarian response, meaning the capacity to respond to FSH, either endogenous or exogenous, such as in ART. This could explain the absence of significant differences in the ovarian reserve, reflected by the AFC count [18,21,32,38,40,41] or AMH levels [18,21,32,38,42], as the surgery does not affect the ovarian cortex and the number of follicles present since embryonic stage remains constant. However, these follicles may not receive as optimal stimulation as before. It is crucial to analyze ovarian reserve and response separately, and this is why the strength of this study was to evaluate only one criterion specific to the final stage follicular response.

The main limitations of our study are its small sample size, its monocentric character, and retrospective design. As the sequence IVF–salpingectomy–IVF is rare, prospective design appears inappropriate. We would have appreciated comparing the number of retrieved oocytes and mature oocytes between the two ovaries as well, but, in our practices, we do not distinguish one side from the other. Furthermore, our study suffers from missing data about the expertise level of the main operator (senior or junior surgeon). On the other hand, the monocentric design offers the opportunity to compare patients who underwent ART procedure with the same protocol, thus limiting the subsequent bias.

Finally, stating that salpingectomy may alter blood flow to the ovaries makes sense, despite the controversial results among the literature. This harmful effect may explain the earlier onset of menopause in patients who have undergone salpingectomy compared to controls [43]. However, there may be surgeries that are more radical than others in terms of altering the mesosalpinx vascularization, which would explain the inter-individual variability in the consequences of this surgery. These findings emphasize the importance of accurately setting surgical indications and timing to perform them. Indeed, the salpingectomy procedure for hydrosalpinx is intended to enhance fertility. However, paradoxically, it may result in reduced fertility in certain cases. It would be interesting to offer a two-stage approach in case of hydrosalpinx diagnosed during ART, especially to patients with poor responder risk factors. The first stage would be IVF with a freeze-all strategy, followed by the salpingectomy surgery. Gynecologists should be aware of this concept, underscoring the significance of surgeons trained in fertility preservation to treat those specific patients. The present results should be interpreted with caution, as a large multicentric retrospective trial with a larger sample size is necessary to obtain more definitive resolution.

## 5. Conclusions

In this retrospective study, we observed a significant decrease in the count of mature follicles in the ipsilateral ovary following salpingectomy in the combined group but not in the sub-group analysis. This decrease may have been due to compromise of the shared vascularization between the ovary and the fallopian tube. These findings the importance of attention to preserving vascularization in the mesosalpinx during salpingectomy. Prospective studies with larger populations are needed to confirm our results.

## Figures and Tables

**Table 1:** Main characteristics. (A). Patients Characteristics. (B). In Vitro Fertilization (IVF) cycle characteristics.

(A)			
	n = 24		
Gestity	1.2 ± 1.4		
Parity	0.2 ± 0.4		
BMI, kg/m^2^	26.1 ± 5.6		
Laterality of unilateral salpingectomy			
Right, n (%)	10.0 (41.7)		
Left, n (%)	14.0 (58.3)		
Type of infertility			
Primary infertility, n (%)	19.0 (79.2)		
Secondary infertility, n (%)	5.0 (20.8)		
Technique of unilateral Salpingectomy			
Laparoscopy	24.0 (100)		
Laparotomy	0.0 (0)		
Smoking history, n (%)	5.0 (20.8)		
Tubal factor, n (%)	11.0 (45.8)		
Endometriosis, n (%)	10.0 (41.7)		
Poor ovarian reserve, n (%)	7.0 (29.2)		
Ovulatory dysfunction, n (%)	4.0 (16.7)		
Male factor, n (%)	7.0 (29.2)		
Interval between surgery and IVF 2, years	1.02 ± 0.9		
(B)			
	IVF 1 (n = 24)	IVF 2 (n = 24)	*p*
Age, years	34.91 ± 4.0	36.8 ± 4.0	0.085
Basal FSH, IU/L	7.7 ± 3.1	8.3 ± 3.1	0.620
Basal AMH, ng/mL	2.7 ± 3.0	1.6 ± 1.5	0.136
Basal AFC, n	12.4 ± 6.4	12.2 ± 7.7	0.793
Total gonadotropins, UI/L	3775.0 ± 2257.5	5364.6 ± 2222.8	0.020
Length, days	10.8 ± 1.4	11.4 ± 2.1	0.363
Antagonist, n (%)	22.0 (91.7)	23.0 (95.8)	
Agonist, n (%)	2.0 (8.4)	1.0 (4.2)	

Note: Values are means ± SD or n (%).

**Table 2:** Sonographic follicular response during ART stimulation, comparing operated side versus control side, before and after unilateral salpingectomy.

	Control Side (n = 24)	Salpingectomy Side (n = 24)	*p*
IVF 1			
Mature follicles (size ≥ 16 mm)	3.79 ± 2.27	4.00 ± 2.43	0.76
Intermediate follicles (13–15.5 mm)	1.65 ± 1.33	1.83 ± 1.78	0.98
Total recruited follicles (≥13 mm)	5.36 ± 2.45	5.74 ± 2.81	0.69
IVF 2			
Mature follicles (size ≥ 16 mm)	5.08 ± 3.89	3.00 ± 2.83	0.048
Intermediate follicles (13–15.5 mm)	1.21 ± 1.48	1.38 ± 1.93	0.90
Total recruited follicles (≥13 mm)	6.23 ± 4.57	4.38 ± 3.93	0.13

Note: Values are means ± SD.

**Table 3:** Subgroup analysis. (A). In the Ectopic Pregnancy Cohort. (B). In the Hydrosalpinx Cohort.

(A)			
	Control Side (n = 15)	Salpingectomy Side (n = 15)	*p*
IVF 1			
Mature follicles (size ≥ 16 mm)	4.40 ± 2.57	4.67 ± 2.77	0.85
Intermediate follicles (13–15.5 mm)	1.29 ± 1.20	1.71 ± 1.82	0.65
Total recruited follicles (≥13 mm)	5.6 ± 2.69	6.29 ± 3.07	0.51
IVF 2			
Mature follicles (size ≥ 16 mm)	5.53 ± 4.00	3.90 ± 3.14	0.22
Intermediate follicles (13–15.5 mm)	1.20 ± 1.27	1.33 ± 1.91	0.60
Total recruited follicles (≥13 mm)	6.73 ± 4.11	5.13 ± 4.26	0.20
(B)			
	Control Side (n = 9)	Salpingectomy Side (n = 9)	*p*
IVF 1			
Mature follicles (size ≥ 16 mm)	2.78 ± 1.09	2.89 ± 1.17	0.85
Intermediate follicles (13–15.5 mm)	2.22 ± 1.39	2.00 ± 1.80	0.72
Total recruited follicles (≥13 mm)	5.00 ± 2.06	4.89 ± 2.26	0.82
IVF 2			
Mature follicles (size ≥ 16 mm)	4.33 ± 3.81	1.67 ± 1.58	0.19
Intermediate follicles (13–15.5 mm)	1.22 ± 1.86	1.44 ± 2.07	0.88
Total recruited follicles (≥13 mm)	5.56 ± 5.43	3.11 ± 3.14	0.26

Note: Values are means ± SD.

## Data Availability

The datasets used and analyzed in this study are available upon reasonable request from the corresponding author.
